# Elevated levels of several chemokines in the cerebrospinal fluid of patients with subarachnoid hemorrhage are associated with worse clinical outcome

**DOI:** 10.1371/journal.pone.0282424

**Published:** 2023-03-09

**Authors:** Pavlos Vlachogiannis, Lars Hillered, Per Enblad, Elisabeth Ronne-Engström

**Affiliations:** Department of Medical Sciences/Section of Neurosurgery, Uppsala University, Uppsala, Sweden; Rigshospitalet, DENMARK

## Abstract

**Background:**

Chemokines are small cytokines that exert chemotactic actions on immune cells and are involved in many inflammatory processes. The present study aims to provide insight in the role of this relatively unexplored family of proteins in the inflammatory pathophysiology of subarachnoid hemorrhage (SAH).

**Materials and methods:**

Cerebrospinal fluid of 29 patients (17 female; mean age 57 years) was collected at days 1, 4 and 10 after SAH, centrifuged and frozen at -70°C. Analysis of 92 inflammation-related proteins was performed using Target 96 Inflammation ^®^ assay (Olink Proteomics, Uppsala, Sweden) based on Proximity Extension Assay technology. The panel included 20 chemokines (CCL2 (or MCP-1), CCL3, CCL4, CCL7 (or MCP-3), CCL8 (or MCP-2), CCL11 (or Eotaxin), CCL13 (or MCP-4), CCL19, CCL20, CCL23, CCL25, CCL28, CXCL1, CXCL5, CXCL6, CXCL8 (or IL-8), CXCL9, CXCL10, CXCL11 and CX3CL1 (or Fractalkine)) that were analyzed for their temporal patterns of expression and compared in dichotomized clinical groups based on World Federation of Neurosurgical Societies (WFNS) admission score and amount of blood on admission CT based on Fisher scale; presence of delayed cerebral ischemia(DCI)/delayed ischemic neurological deficit (DIND); and clinical outcome based on Glasgow Outcome Scale. Protein expression levels were provided in output unit Normalized Protein Expression (NPX). ANOVA models were used for statistical analyses.

**Results:**

Four temporal patterns of expression were observed (i.e., early, middle, late peak and no peak). Significantly higher day 10 mean NPX values were observed in patients with poor outcome (GOS 1–3) for chemokines CCL2, CCL4, CCL7, CCL11, CCL13, CCL19, CCL20, CXCL1, CXCL5, CXCL6 and CXCL8. In the WFNS 4–5 group, CCL11 showed significantly higher day 4 and day 10 mean NPX values and CCL25 significantly higher day 4 values. In patients with SAH Fisher 4, CCL11 showed significantly higher mean NPX values on days 1, 4 and 10. Finally, patients with DCI/DIND had significantly higher day 4 mean NPX values of CXCL5.

**Conclusion:**

Higher levels of multiple chemokines at the late stage of SAH seemed to correlate with worse clinical outcome. A few chemokines correlated with WFNS score, Fisher score and occurrence of DCI/DIND. Chemokines may be useful as biomarkers for describing the pathophysiology and prognosis of SAH. Further studies are needed to better understand their exact mechanism of action in the inflammatory cascade.

## Introduction

Subarachnoid hemorrhage (SAH) is a life-threatening stroke associated with high mortality and morbidity. It tends to affect younger people compared to other types of strokes and carries a significant socioeconomic burden since almost 50% of survivors suffer from long term neurological and psychological deficits and decreased quality of life. The most common cause in 85% of the cases is rupture of intracranial aneurysm [1, 2].

The inflammatory response that follows SAH has been the focus of extensive research in the past decades with numerous studies addressing different cellular and molecular mechanisms that seem to be involved [3–6]. Shortly after aneurysm rupture and the deposition of blood in the subarachnoid space innate immune cells that migrate from the periphery into the central nervous system (CNS) (such as neutrophils and monocytes) and those that reside within the CNS (such as microglia) are activated. At the same time a plethora of inflammatory mediators such as cytokines are upregulated and released in the cerebrospinal fluid (CSF) and plasma [7, 8]. A cascade of inflammatory events is thus triggered and maintained throughout the acute phase of SAH that can be roughly divided into two periods, Early Brain Injury (EBI) and Delayed Cerebral Ischemia (DCI). EBI refers to the events occurring between 0–72 h from the aneurysm rupture while DCI concerns pathophysiological changes that emerge between days 4–14 [9, 10]. The latter was traditionally attributed to cerebral vasospasm (CV), but recent evidence suggests that it is a multifactorial phenomenon where CV is only partially responsible [11].

In a recent explorative study we defined different temporal patterns of 64 inflammatory proteins in CSF after spontaneous SAH [12]. In the present study we sought to analyze more in detail the clinical significance of a large subgroup of these inflammatory proteins–the chemokines. Chemokines (*chemo*-tactic cyto-*kines*) are a family of approximately 50 structurally and functionally related small proteins that mediate trafficking of immune cells [13]. Traditionally, chemokines were considered to mediate local inflammatory response against various infections or trauma, but recent evidence suggests that they are involved in numerous physiological and pathological conditions including homeostasis, development, autoimmune and inflammatory diseases and cancer [14]. The role of chemokines in post SAH inflammation has not been fully elucidated. Recent clinical and experimental studies have pointed to single chemokines and/or their receptors as potential predictors of vasospasm and outcome as well as possible targets for intervention [15–20].

In the present study the expression levels of multiple chemokines were measured simultaneously in the CSF of 29 patients with SAH using Target 96 Inflammation ^®^ assay (Olink Proteomics, Uppsala, Sweden) based on Proximity Extension Assay (PEA) technology [21, 22]. Different temporal patterns of expression were noticed, and the chemokines were grouped accordingly. The results were further analyzed for potential correlations with clinical parameters in dichotomized patient groups based on clinical status and amount of blood on admission, occurrence of CV and functional outcome 1 year after SAH. The aim of the study was to report on new chemokines that had not been studied in SAH context previously and to add to the already existing literature on other chemokines such as CCL2 and CXCL8 that have long been the focus of research [23].

## Materials and methods

### Patients

The study cohort consisted of 29 patients (17 females and 12 males; mean age 57 years) with spontaneous SAH that were admitted to the Neurointensive Care Unit of Uppsala University Hospital within 24h from ictus. Median World Federation of Neurosurgical Societies (WFNS) score on admission was 4 [24]. Diagnosis was made by brain CT scans that were classified according to Fisher scale (median 4) [25]. Cerebral vasospasm was defined clinically (i.e., not on angiography) as delayed focal ischemic neurological deficit (DIND) and/or decrease in the level of consciousness on the Glasgow coma scale not attributable to new hemorrhage or hydrocephalus and/or as delayed cerebral ischemia (DCI) seen on CT scan. Patients with only ischemic lesions on CT scan without clinical signs of focal neurological deficit were also defined as having “cerebral vasospasm”; hence, the two terms DIND and DCI were used indiscriminately in the present study and their occurrence was recorded and treated accordingly. A research nurse recorded the patients’ functional outcome based on Glasgow Outcome Scale one year post ictus through telephone interviews [26].

### Dichotomized patient groups

The patient cohort was dichotomized in groups based on WFNS score on admission (1–3 = 13 patients vs 4–5 = 16 patients); Fisher grade on initial CT scan (grade 1–3 = 12 patients vs grade 4 = 17 patients); occurrence of DCI/DIND at any time point during the observation period (no = 16 patients vs yes = 13 patients); and favorable vs unfavorable functional outcome (GOS 4–5 = 9 patients vs GOS 1–3 = 20 patients).

### Sample collection and analysis

External ventricular drain (EVD) was placed in all 29 patients on admission within 24 h from ictus. CSF samples were obtained at the time of surgery and centrifuged directly after, and supernatant fluid was stored in -70°C (day 1 samples). Additional CSF samples were collected through the EVD on days 4 and 10 during the Neurointensive Care Unit stay and the same procedure with centrifugation and freezing was followed. Patients whose EVD was discontinued for clinical reasons before day 10 and patients in deep coma with bilaterally dilated and fixed pupils who were deemed terminally ill from the bleeding were not considered eligible to participate.

Multiplex Proximity Extension Assay (PEA) technology was used for the analysis of the collected samples [21]. For this purpose, Target 96 Inflammation ^®^, a multiplex assay panel commercially available by Olink Proteomics, Uppsala, Sweden, was employed (https://www.olink.com). The panel includes 92 inflammatory biomarkers that can be analyzed simultaneously in very small samples (i.e., 1 μL) and with high specificity due to PEA technology [22]. PEA technology can be summarized as follows: 1 μL of CSF is incubated with a matched pair of two oligonucleotide-conjugated antibodies (“probes”) that bind specifically to each target protein present in the sample. The affinity bindings between the antibodies bring the probes in proximity allowing for their extension by enzymatic DNA polymerization. The DNA sequence unique for each protein that forms this way can then be detected, amplified and quantified by real-time quantification Polymerase Chain Reaction (qPCR). The number of qPCR cycles required for detection is related to the concentration of the protein in the sample. The raw Ct values are then normalized against negative- and spiked-in controls to achieve relative quantification values that are provided in output unit Normalized Protein Expression (NPX) in log2 scale which correlates positively with the actual protein concentration in the sample. Limit of detection (LOD) is determined for each biomarker based on the negative controls analyzed in each run.

### Chemokines

The aim of the present study was a comprehensive analysis of chemokines regarding their temporal patterns and correlations with clinical parameters. To that purpose the 20 chemokines included in the panel were isolated and further analyzed. In detail, twelve members of the “C-C family”, i.e., CCL2, CCL3, CCL4, CCL7, CCL8, CCL11, CCL13, CCL19, CCL20, CL23, CCL25 and CCL28; seven members of the “C-X-C family”, i.e., CXCL1, CXCL5, CXCL6, CXCL8, CXCL9, CXCL10 and CXCL11; and finally, the only member of the “C-X3-C” family CX3CL1. Notably, on the Olink Proteomics web site some of the included chemokines are listed with an alternative first name, i.e., CCL2 as MCP1, CCL7 as MCP3, CCL8 as MCP2, CCL11 as Eotaxin, CCL13 as MCP4, CXCL8 as IL-8 and CX3CL1 as Fractalkine.

### Statistics

Appropriate Analysis of Variance (ANOVA) models depending on the normality of data distribution and statistical assumptions were used for statistical analyses. Repeated measures ANOVA was used when data were normally distributed (tested with histograms and Shapiro-Wilk’s test) and the assumptions of homogeneity of variances (tested with Levene’s test) and compound symmetry (tested with Mauchley’s sphericity test) were met. Fisher LSD test was used for post-hoc analyses when indicated. Nonparametric ANOVA models were used when data were not normally distributed and/or the above-named assumptions were not met. More specifically, Friedman’s ANOVA was used for comparisons of means between days 1, 4 and 10 for each chemokine in order to determine statistically significant peaks and trends between time points throughout the observation period. In those cases where the variance was found to differ significantly, further pairwise comparisons were done with Wilcoxon’s test (i.e. day 1 vs day 4, day 4 vs day 10 and day 1 vs day 10). Kruskal-Wallis ANOVA was used to compare mean NPX values between the different clinical groups on each time point. Results were considered significant at the p<0.05 level. All statistical analyses and graphical presentations were performed using the Statistica^®^ software (Stat Soft, Inc., Tulsa, OK, USA).

### Ethics

The study was conducted in accordance with Declaration of Helsinki for human studies and approved by Uppsala University Ethics Committee. All participants or their next of kin gave written consent for participation in the study.

## Results

Most of the samples were successfully analyzed. Only 3 chemokines showed single NPX values under LOD. CCL11 had one missing value on day 4; CCL13 had 17, 13 and 4 missing values on days 1, 4 and 10, respectively; finally, CCL25 had 3 and 1 missing values on days 4 and 10 respectively.

**Fig 1:**
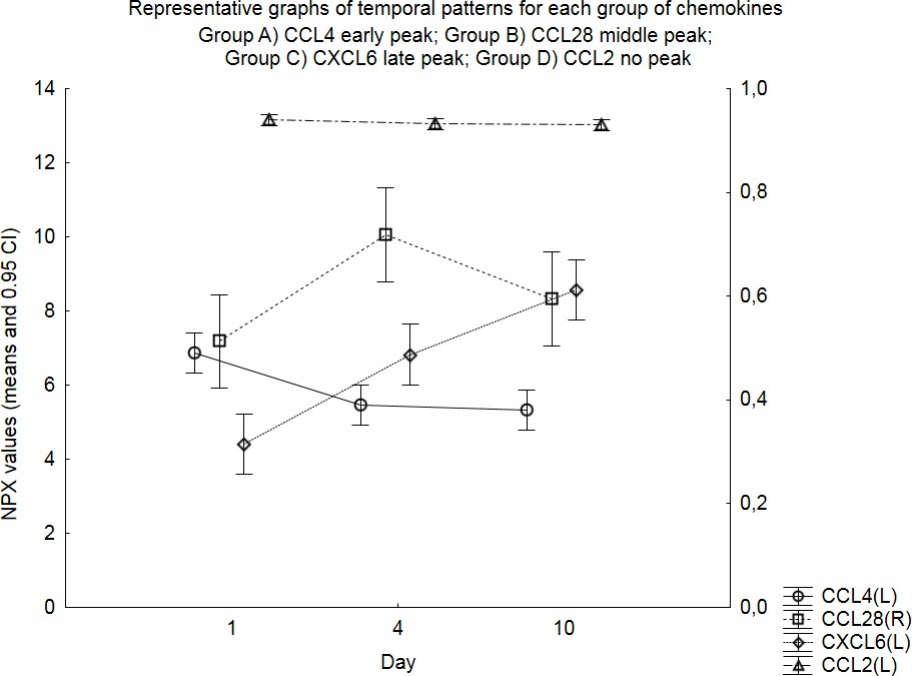
Representative graphs of temporal pattern of expression for each group of chemokines: Four different temporal patterns of expression were noticed, and the chemokines were grouped accordingly. Representative graphs for each chemokine group are illustrated with their means and 0.95 CI for each day. More specifically, CCL4 is shown for Group A with its early peak and decreasing trend; CCL28 was the only chemokine of Group B with a significant middle peak on day 4; CXCL6 with its increasing trend and late peak is shown for Group C; and finally, CCL2 is representing Group D with stable values throughout the observation period. Note that NPX values of CCL4 (representing Group A), CXCL6 (Group C) and CCL2 (Group D) are shown on the left Y-axis (L) whereas values of CCL28 (representing Group B) are shown on the right Y-axis (R).

Mean NPX values with ±0.95 confidence intervals for each day were plotted in graphs for all chemokines and four distinguished temporal patterns of expression were noticed (Fig 1). Four chemokines (CCL3, CCL4, CCL11 and CCL20) showed early peak and decreasing values (Group A). CCL28 showed a statistically significant middle peak (Group B). The majority of the chemokines (i.e., 11/20; notably, almost all members of the C-X-C chemokine family except CXCL5) showed increasing trends throughout the observation period and late peaks on day 10 (Group C). Finally, chemokines CCL2, CCL25, CXCL5 and CX3CL1 did not show any specific pattern and had rather stable values throughout the observation period (Group D). Statistical analysis disclosed significant between-days differences for some chemokines as shown in Table 1.

**Table 1:** Between-days comparisons of mean NPX values (+/- 0.95 CI) for each chemokine.

	D1 mean NPX	D1 vs D4	D4 mean NPX	D4 vs D10	D10 mean NPX	D1 vs D10
**CCL2**	13.15 (13.04–13.26)		13.04 (12.86–13.23)		13.02 (12.85–13.18)	
**CCL3**	3.83 (3.14–4.52)		3.21 (2.69–3.73)		3.19 (2.81–3.56)	
**CCL4**	6.86 (6.14–7.59)	**	5.46 (4.93–6)		5.33 (4.96–5.69)	**
**CCL7**	5.29 (4.65–5.93)	*	6.27 (5.60–6.94)		6.65 (5.92–7.39)	**
**CCL8**	6.98 (6.34–7.62)	**	8.13 (7.42–8.84)		8.88 (8.24–9.52)	**
**CCL11**	3.21 (2.76–3.67)	**	2.36 (2.15–2.58)		2.47 (2.26–2.68)	**
**CCL13**	0.75 (0.54–0.95)		0.66 (0.57–0.76)		1.14 (0.9–1.39)	
**CCL19**	7.74 (7.27–8.21)	**	10.38 (9.66–11.1)	*	11.08 (10.44–11.72)	**
**CCL20**	6.21 (5.25–7.17)		5.97 (5.19–6.75)		5.58 (4.90–6.26)	
**CCL23**	3.86 (3.38–4.34)	**	5.89 (5.27–6.51)	**	7.26 (6.65–7.86)	**
**CCL25**	1.68 (1.34–2.03)		1.34 (1.16–1.52)		1.56 (1.28–1.85)	
**CCL28**	0.51 (0.38–0.64)	**	0.71 (0.65–0.78)	*	0.59 (0.52–0.66)	
**CXCL1**	7.44 (6.81–8.06)	*	8.80 (7.98–9.62)	*	9.55 (8.79–10.32)	**
**CXCL5**	8.35 (7.59–9.11)		8.52 (7.81–9.23)		8.59 (7.89–9.30)	
**CXCL6**	4.41 (3.74–5.07)	**	6.81 (5.85–7.77)	**	8.57 (7.68–9.45)	**
**CXCL8**	12–80 (12.3–13.29)		12.89 (12.25–13.52)	*	13.34 (12.73–13.95)	
**CXCL9**	3.38 (2.96–3.80)	**	4.68 (4.15–5.22)	**	5.86 (5.2–6.52)	**
**CXCL10**	7.7 (7.04–8.50)	**	10.44 (9.67–11.22)		10.92 (10.33–11.52)	**
**CXCL11**	2.84 (2.41–3.28)	**	3.98 (3.40–4.56)		4.30 (3.83–4.76)	**
**CX3CL1**	2.98 (2.67–3.29)		3.31 (2.96–3.67)		3.45 (3.14–3.77)	

Appropriate ANOVA models were used for the comparisons, i.e., Repeated-Measures ANOVA and Fisher’s LSD test for post-hoc analyses when data were normally distributed, and statistical assumptions of homogeneity of variances and compound symmetry were met; and Friedman ANOVA with Wilcoxon’s test when nonparametric statistics were deemed necessary. Statistically significant differences between days are marked with asterisks

(* p<0.05

** p<0.01).

**Fig 2:**
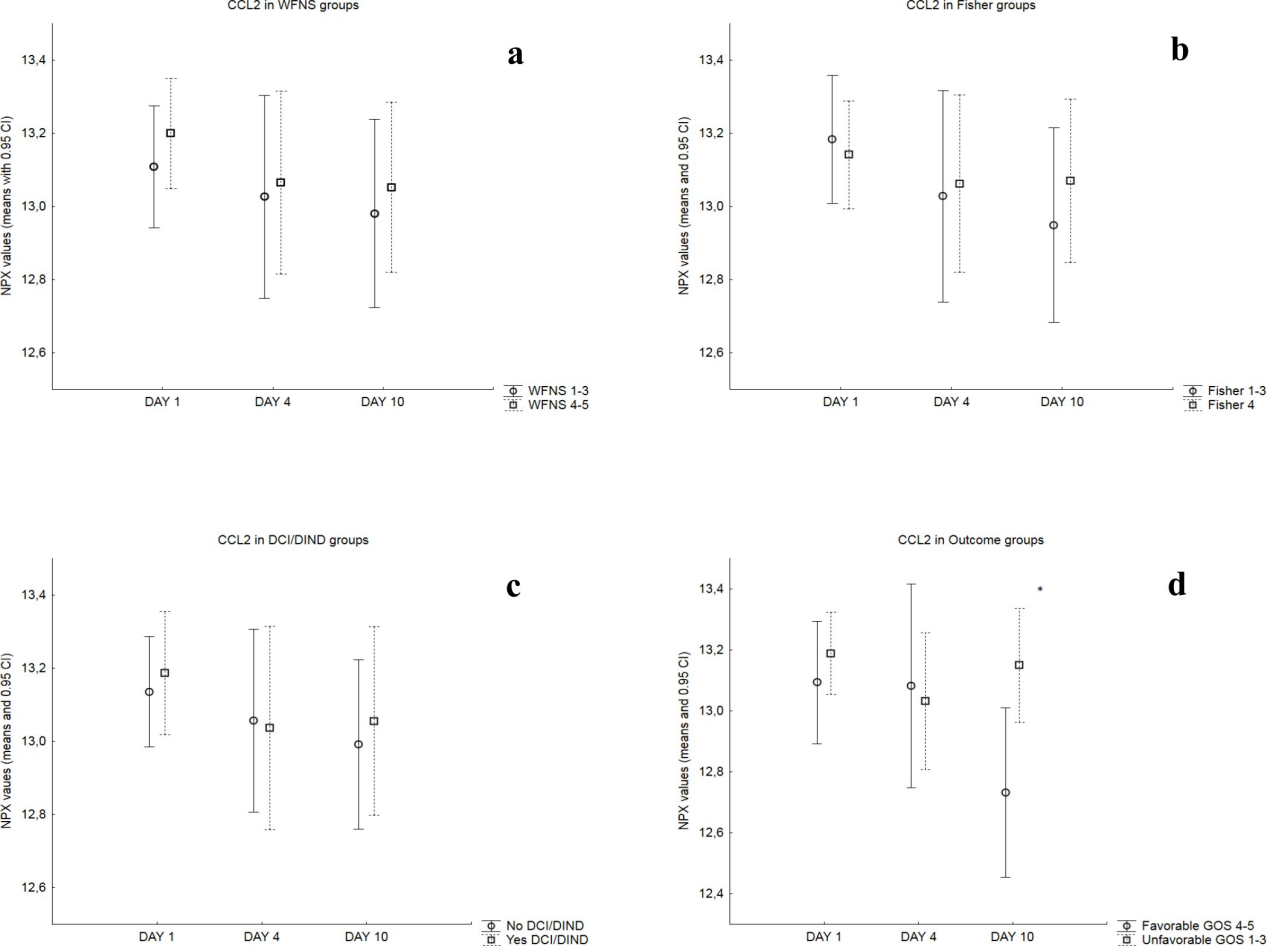
Comparisons of CCL2 NPX values in patient groups: Mean NPX values and 0.95 CI for each day in dichotomized patient groups based on a) WFNS score on admission (*top left*), b) Fisher scale of admission CT (*top right*), c) occurrence of DCI/DIND (*bottom left*) and d) clinical outcome (*bottom right*). The asterisk on the bottom right image marks significantly higher day 10 mean NPX values for patients with unfavorable outcome.

**Fig 3:**
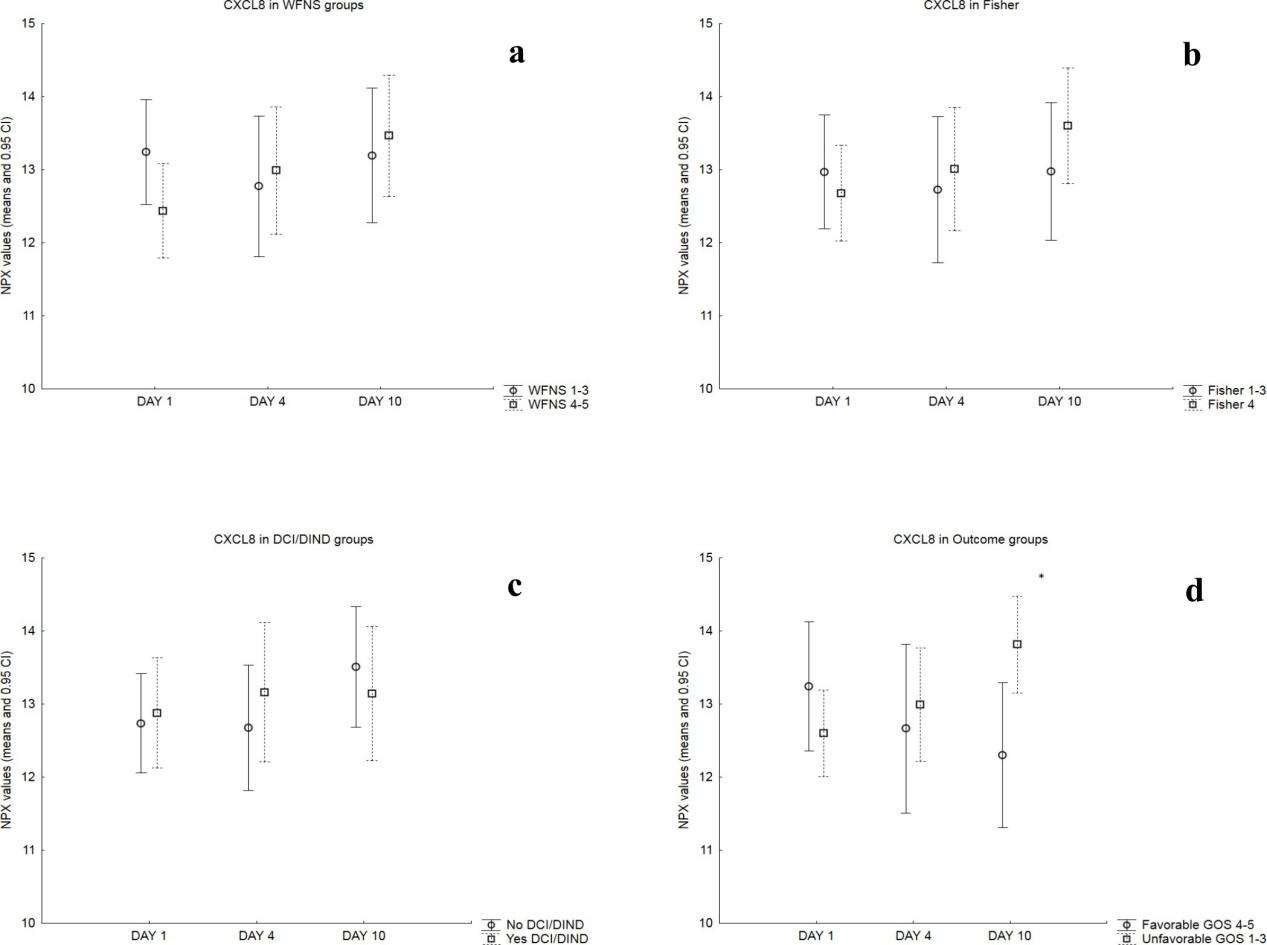
Comparisons of CXCL8 NPX values in patient groups: Mean NPX values and 0.95 CI for each day in dichotomized patient groups based on a) WFNS score on admission (*top left*), b) Fisher scale of admission CT (*top right*), c) occurrence of DCI/DIND (*bottom left*) and d) clinical outcome (*bottom right*). The asterisk on the bottom right image marks significantly higher day 10 mean NPX values for patients with unfavorable outcome.

**Fig 4:**
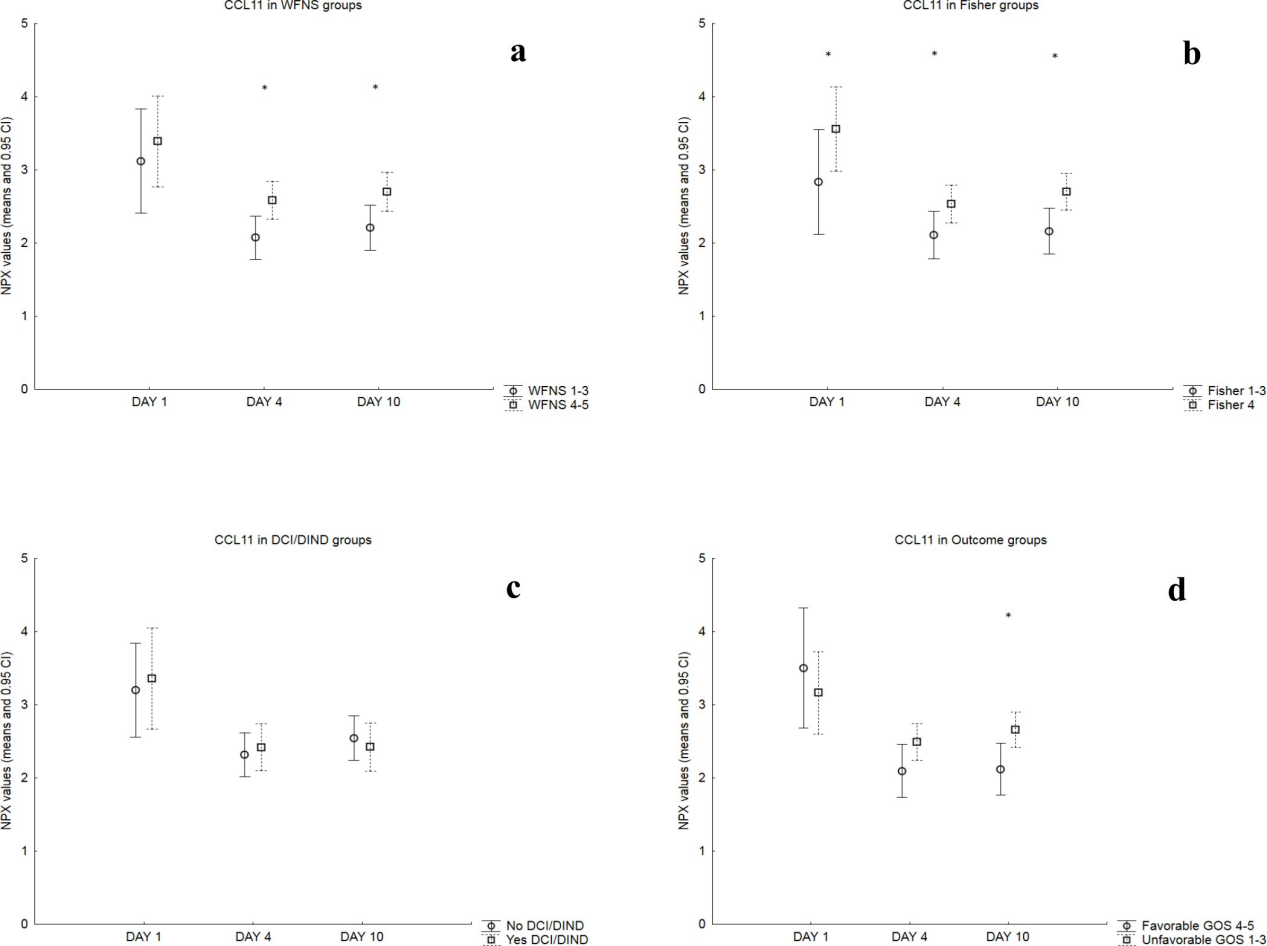
Comparisons of CCL11 NPX values in patient groups: Mean NPX values and 0.95 CI for each day in dichotomized patient groups based on a) WFNS score on admission (*top left*), b) Fisher scale of admission CT (*top right*), c) occurrence of DCI/DIND (*bottom left*) and d) clinical outcome (*bottom right*). Asterisks on the top left and right images as well as the bottom right image mark statistically significant differences in mean NPX values for the respective day in each group.

Mean NPX values for each day were then compared in dichotomized patient groups as described in the Methods section and the results are summarized in Table 2. Figs 2–4 illustrate these comparisons in detail for chemokines CCL2 and CXCL8 (that are the most well studied chemokines in the literature) as well as for CCL11 (that provided the most findings in the present study). Patients with WFNS Score = 4–5 showed significantly higher day 4 and day 10 values for chemokine CCL11 and day 4 values for chemokine CCL25, compared to patients with WFNS Score = 1–3. Significantly higher NPX values for CCL11 were a consistent finding in all time points throughout the observation period for patients with Fisher 4 SAH, compared to Fisher 1–3. Mean day 4 NPX values of CXCL5 were significantly higher in patients with DCI/DIND compared to those without. Finally, patients with unfavorable outcome GOS = 1–3 had significantly higher day 10 NPX values for chemokines CCL2, CCL4, CCL7, CCL11, CCL13, CCL19, CCL20, CXCL1, CXCL5, CXCL6 and CXCL8.

**Table 2:** Comparison of chemokine expression levels in dichotomized patient groups.

DICHOTOMISED CLINICAL GROUPS	CHEMOKINES WITH SINGNIFICANTLY HIGHER VALUES (& SPECIFIC DAYS)
**WFNS 4–5 (n = 16)**	**CCL11** (D4 & D10)
	**CCL25** (D4)
**Fisher 4 (n = 17)**	**CCL11** (D1, D4 & D10)
**DCI/DIND (n = 13)**	**CXCL5** (D4)
**GOS 1–3 (n = 20)**	**CCL2** (D10)
	**CCL4** (D10)
	**CCL7** (D10)
	**CCL11** (D10)
	**CCL13** (D10)
	**CCL19** (D10)
	**CCL20** (D10)
	**CXCL1** (D10)
	**CXCL5** (D10)
	**CXCL6** (D10)
	**CXCL8** (D10)

The patient cohort was dichotomized in groups based on WFNS and Fisher score on admission, occurrence of DCI/DIND and functional outcome 1 year post ictus based on GOS. NPX values of all chemokines were then compared between patient groups for each day. Appropriate ANOVA models were used for the comparisons as described in the Methods section and the results are summarized in the table. Significantly higher expression levels (p<0.05) were found in patients with WFNS 4–5 for chemokines CCL11 (day 4 and day 10) and CCL25 (day 4); in patients with Fisher 4 for CCL11 (all 3 days); in patients with DCI/DIND for CXCL5 (day 4); and finally in patients with unfavorable outcome GOS = 1–3 for CCL2, CCL4, CCL7, CCL11, CCL13, CCL19, CCL20, CXCL1, CXCL5, CXCL6 and CXCL8 (day 10 values for all chemokines).

## Discussion

The main finding of this study is that several chemokines may be used as outcome predictors after SAH since their late peaks at day 10 seem to correlate with worse clinical outcome at 1 year after the bleeding. Some of these chemokines have been previously reported to correlate with outcome and other clinical parameters while others have not been studied in SAH context before.

Chemokine CCL2 (or Monocyte chemoattractant protein 1) is a potent chemoattractant for circulating blood monocytes and a well-studied chemokine in SAH. In the present study, CCL2 showed an intense activation pattern with high mean NPX values throughout the whole observation period, although without variations between different days (Table 2; Fig 1). Moreover, day 10 values were significantly higher in patients with unfavorable outcome (Fig 2). In line with our findings, higher serum levels of CCL2 were described to correlate with worse clinical outcome in studies by Kim et al in 2008 and Ahn et al in 2019 [17, 20]. Mohme et al even demonstrated that monocyte accumulation and activation in the CSF secondary to early peak of CCL2 correlated with DCI (which was the main topic of the study) but also worse clinical outcome, thus highlighting this chemokine’s role as an important mediator of the innate inflammatory response in SAH patients [27].

Another recurrent chemokine in SAH literature is CXCL8 (or Interleukin-8) that is primarily involved in the chemotaxis of neutrophils. A consistent finding is the elevated levels in the CSF and plasma of SAH patients compared to healthy controls even without reporting on associations with outcome or other clinical parameters [28, 29]. Late peak of CXCL8 on day 10 correlated with worse clinical outcome 1 year after SAH in the present study (Fig 3). Similarly, Nakahara et al reported higher CSF levels of IL-8 (among other cytokines) in patients with worse clinical outcome 3 months post SAH [30]. However, plasma IL-8 was among the cytokines reported not to correlate with outcome and DCI in SAH patients in a recent study by Rasmussen et al [31].

Chemokine CCL11 (also known as Eotaxin) provided the most striking findings in the study (Fig 4). Significantly higher NPX values were noticed on days 4 & 10 in patients with WFNS 4–5 and days 1, 4 & 10 in patients with Fisher 4 bleeding. Finally, higher day 10 values correlated with worse clinical outcome. CCL11 has not been studied extensively in SAH patients as the only report comes from Savarraj et al who found elevated serum levels early after SAH (i.e., <24h) in patients with higher clinical severity at admission (Hunt and Hess 4) [32]. A significant early peak in CSF was noticed in our study (Fig 1, Table 2) but, in contrast to Savarraj’s study, no difference in the WFNS 4–5 group on day 1 values was found (Fig 4). CCL11 is mainly implicated in the chemotactic migration of eosinophils and basophils to peripheral tissues through interaction with CCR3 and CCR2 receptors and these cells have not been reported to play an important role in the SAH pathophysiology but if similar results are reported from other studies this could represent an interesting area for further research [13].

Other chemokines not included in the present panel have also been associated with poor outcome after SAH. Higher serum levels and lower CSF levels of chemokine CCL5 were found in SAH patients with good clinical outcome at discharge (modified Rankin scale 0–2 and/or Glasgow Outcome Scale 4–5) by Chaudhry et al [16]. Moreover, elevation of serum CXCL12 was found to be associated with worse clinical outcome and bleeding severity [19].

An important point that needs to be raised is the possible effect of bacterial meningitis/ventriculitis on the levels of the inflammatory markers especially on Days 4 and 10. The number of patients with culture-verified bacterial meningitis/ventriculitis during the observation period were 6/29. The diagnosis was verified on day 2 (one patient); day 4 (one patient); day 6 (two patients); day 8 (one patient); and day 10 (one patient) after SAH and EVD insertion. The number of patients treated with antibiotics at some point during the EVD treatment due to suspected meningitis/ventriculitis was 24/29. Whenever cultures were negative after some days the antibiotics were discontinued or adjusted in doses and schemes as to address other infections that might had emerged in the meantime (such as pneumonia). No patient received any other anti-inflammatory treatment, such as steroids.

Further analysis of chemokine levels based on the occurrence of bacterial meningitis/ventriculitis was not performed for several reasons. Firstly, the presence of the chemical meningitis/ventriculitis in SAH patients could make it difficult to isolate the effect of a possible concurrent bacterial infection. Moreover, the fact that antibiotics were commonly given “prophylactically” directly after suspected bacterial infection could eradicate a possible bacterial contamination early before it developed to a full-blown meningitis/ventriculitis. Finally, the number of patients with verified meningitis was small and did not allow for adequate comparisons.

### Limitations

The small number of patients represents a weakness of the study. Comparison with healthy controls could strengthen the results. Moreover, the PEA method in its current form does not provide absolute protein concentrations which does not allow for direct comparisons of absolute values. However, in combination with cerebral microdialysis it allows for multiple comparisons of relative quantification as well as temporal patterns. The lack of further analysis of chemokine levels based on the occurrence of meningitis/ventriculitis may be considered a limitation. Finally, bioinformatics analysis could possibly illustrate how the different chemokines correlate to each other and allow for further studies aiming at the identification of potential biomarkers predictive of outcome and other clinical parameters of interest, such as DCI.

## Conclusions

Late peaks of several chemokines seem to correlate with worse clinical outcome post SAH. Further studies in this promising and relatively unexplored family of inflammation-related proteins may lead to identification of biomarkers for prognostication of the disease severity and outcome and potentially contribute to tailored treatment.
